# Security Evaluation of Companion Android Applications in IoT: The Case of Smart Security Devices

**DOI:** 10.3390/s24175465

**Published:** 2024-08-23

**Authors:** Ashley Allen, Alexios Mylonas, Stilianos Vidalis, Dimitris Gritzalis

**Affiliations:** 1 Cybersecurity and Computing Systems Research Group, Department of Computer Science, University of Hertfordshire, Hatfield, AL10 9AB, UK; a.allen3@herts.ac.uk (A.A.);; 2 Department of Informatics, Athens University of Economics and Business (AUEB), 76 Patission Ave., GR-10434 Athens, Greece; dgrit@aueb.gr

**Keywords:** cybersecurity, smart home, IoT, Android, software development, SAST

## Abstract

Smart security devices, such as smart locks, smart cameras, and smart intruder alarms are increasingly popular with users due to the enhanced convenience and new features that they offer. A significant part of this convenience is provided by the device’s companion smartphone app. Information on whether secure and ethical development practices have been used in the creation of these applications is unavailable to the end user. As this work shows, this means that users are impacted both by potential third-party attackers that aim to compromise their device, and more subtle threats introduced by developers, who may track their use of their devices and illegally collect data that violate users’ privacy. Our results suggest that users of every application tested are susceptible to at least one potential commonly found vulnerability regardless of whether their device is offered by a known brand name or a lesser-known manufacturer. We present an overview of the most common vulnerabilities found in the scanned code and discuss the shortcomings of state-of-the-art automated scanners when looking at less structured programming languages such as C and C++. Finally, we also discuss potential methods for mitigation, and provide recommendations for developers to follow with respect to secure coding practices.

## 1. Introduction

Internet of Things (IoT) devices are becoming pervasive in both domestic and business environments, serving a variety of purposes. IoT technology allows the user to perform remote actions, network devices together, and receive alerts of events of interest. Of these smart security devices, one of the most popular is the smart lock. These devices are drop-in replacements for traditional mechanical locks that add smart capabilities, such as remote unlocking, timed access, and software key sharing. Previous work such as [1] has looked at vulnerabilities in these devices, and in the implementation of communication protocols such as Bluetooth Low Energy (BLE), Wi-Fi, RFID, and Zigbee. An interested reader can look to [2,3,4,5] for current research into Bluetooth security, and to [6,7,8] for research into RFID security. Furthermore, as IoT devices are the “lowest hanging fruit in cybersecurity”, there have been several cases in the current threat landscape (e.g., Mirai botnet), where these devices have been compromised and used by threat actors to mount other attacks, such as distributed denial of services (DDoS) [9].

Smart security devices often have an associated software component, separate to the physical component, in the form of mobile phone companion apps. These companion applications run on the user’s mobile phone and provide enhanced functionality. This can range from auto-locking or timed locking and unlocking, through to the sharing of temporary codes with other people. As these companion applications manage devices that protect physical assets, developers should ensure that they are free from vulnerabilities and coded with security in mind. Previous work such as [10,11] discuss ways of ensuring that applications are written in a secure fashion. However, other recent research such as [12,13] indicates that vulnerabilities are still found across the majority of tested apps. This presents a significant risk to the end user, especially where these smart devices are used in situations where financial loss or physical injury may occur.

Furthermore, with the popularity of “direct to consumer” marketplaces and the ability for sellers to sell their products at low cost via established channels, such as eBay and Amazon, the barrier to entry has been lowered considerably. This means that “lesser known” brands can compete directly with those bearing an established name, such as Yale or Chubb. The benefit of this is enhanced consumer choice. This comes with a potential cost, in that companies selling under “white label” or “disposable” brand names may feel less inclined to support their products. As part of the testing performed, we have compared the results obtained from traditional brands and their “lesser known” counterparts.

Secure coding metrics have existed for many years. Bodies such as OWASP [14] and security vendors such as Veracode [15] describe factors, such as code coverage, vulnerability density, false positive rates, as methods for determining the “quality” of written code. When one is using the companion applications to these security devices, they need to be confident that their developers have considered their security, and thus they are not exposed to cyber attacks. Previous research from [16] discusses the wide range of methodologies open to developers to ensure that their code is secure. In addition, AI-enhanced coding tools are now also available to developers and work in [17] indicates that these help to produce more secure code.

In this paper, we assess 54 smart security companion applications and present our results with regards to common vulnerabilities discovered in their implementation. To do so, we propose and use 13 different metrics to measure the security performance of the apps. In summary, this paper makes the following contributions:

- We provide a comparison of the number of common vulnerabilities found in 54 Android companion applications of smart security devices, such as smart lock, smart camera, and smart alarm. To identify these vulnerabilities, we use both commercial and open-source automated static application security testing (SAST) software used in SecDevOps and manual testing.
- We uncover that every tested companion application contains at least one vulnerability in scope of our analysis. Several applications contain critical vulnerabilities that have been known for multiple years. We further identify and discuss concerning practices used by developers of the companion apps, which could adversely impact the user’s security and impair privacy, as well as potentially break relevant laws, such as General Data Protection Regulation (GDPR).
- We discuss remediation steps and recommendations that will allow developers to create more secure apps. We also discuss the steps that users can take to protect themselves when using these apps.

The rest of the paper is structured as follows: Section 2 includes background and related work. Section 3 describes our methodology and Section 4 presents our results. Section 5 includes a discussion before the paper concludes in Section 6. 

## 2. Background

Significant literature from the past 15 years exists with respect to Android application testing in general. This section includes relevant work from the past five years. In their 2021 paper [18], the authors conduct a review of previous literature and produce a threat taxonomy for Android apps. This presents researchers with a coherent methodology for assessing distinct types of application threats. The authors of [19] discuss the varying types of mobile application testing tools available, although their coverage of security tools is weak. Work in [20] also discusses software analysis of a significant (i.e., more than 1000) number of Android apps, focusing on code quality and application design. The authors also discuss the findings with a focus group of five subject matter experts. Their results suggest that most Android applications are poorly written, and existing tests are weak at finding code defects during the design and quality assurance phases of development. Further systematic study of previous literature is made in [21], which discusses the range of tools available for analysing vulnerabilities in web applications. The range of vulnerabilities discussed in the OWASP Mobile Top 10 [22] has a significant overlap with those discussed in the OWASP Web Application Top 10 [23] and so attacks and defences discovered for one domain will have validity in the other. Similar studies can be found in [24,25]. The work of [26] looks at Android application analysis using MobSF and finds similar coding issues to the ones we have found. The authors of [27] also use MobSF to investigate the coding standards found inside an Indonesian eGovernment app. Additionally, the work of [28,29] presents static analysis studies similar to ours relating to hotel booking applications and wearable health tech companion applications, respectively.

Much of the relevant literature focuses on the Android permissions ecosystem and developers’ overuse of dangerous permissions. In [30], the authors present a comprehensive analysis of the permissions system since its introduction in 2008. In reviewing the changes in the permissions requested by several applications over a period of years, the authors have identified significant increases in requested privileges. In one case, a growth of more than 70% was identified. Whilst the increased permission requests may be the result of the addition of new features to the app, it may also indicate a more relaxed attitude to producing tightly defined code. Users are also often confused by the permissions that are being asked of them and may ignore them, as discussed in [31,32]. The authors of [33] have created a tool that automatically checks applications for permission-related vulnerabilities. Again, this suggests that application developers might not be paying enough attention to the output of their security tooling, if it is being used at all. The works of [34,35] discuss issues relating to runtime permissions, indicating that many Android applications suffer from vulnerabilities caused by overly permissive permission models. More recently, [36] looks at hashing the AndroidManifest.xml file, which is used as a signature to quickly verify whether the file has been modified. Finally, the authors of [37] discuss the use of machine learning techniques, specifically XGBoost, to perform granted Android permission analysis over a large sample set.

In [38], the authors discuss the prevalence of weak application configurations relating to the enforcement of HTTPS. Their paper indicated that only 13% of applications were taking advantage of options available to prevent data transfer via a weak method, such as HTTP. Scanning of the same dataset at a later date, after developers had released new versions of the same apps, improved this percentage, but it indicates that many developers are either unaware of enhanced security configurations, or are under no pressure to use them. Finally, the authors of [39] discuss using unsupervised machine learning to identify incorrect usage of cryptographic functions across a real-world dataset.

Third-party libraries, i.e., those which are not part of the core Android source code, are an important part of many Android applications. However, using them presents developers with challenges around auditing them for malicious or unwanted behaviour, as well as potential supply chain issues. The authors of [40] introduce their tool for identifying these libraries in published applications. This technology allows a more accurate vulnerability scanning of applications, but it can also aid malicious actors with the identification of potential attack vectors. Similarly, work in [41] reviews available third-party library detection tools, as well as a roadmap for the creation of more accurate scanners from the combination of more than one tool. Work in [42] discusses one specific scanner and presents an evaluation of its detection capability. Further examples of third-party library detection methods are also given in [43].

In their 2020 paper [44], the authors investigate collaborative bug finding and propose identifying classes of vulnerabilities by investigating similar apps. Similarly, the work in [45] focuses on triaging vulnerabilities and vulnerabilities using Bayesian graphs. Additional work focusing on the detection of multiple classes of vulnerabilities and vulnerabilities can be found in [46,47].

Related works in the literature discuss security frameworks and strategies for conducting security-based assessment. A 2022 paper by [48] discusses the usage of formal methodologies in security research, noting in the conclusion that they provide “evidence of the presence, not the absence of flaws”. This indicates that the task of finding *new* flaws (our emphasis) is best suited to experimental methods, whilst formal methodologies are best suited to catching security vulnerabilities of a known and classified type. The authors of [49] discuss a new, SLA-based, secure by design methodology. By using threat modelling and risk management processes, they aim to reduce the number of vulnerabilities that reach the end product. The authors in [50] discuss SAST tooling, providing detail on 7 tools chosen from a field of 161. Their work indicates that performance against synthetic benchmarks outperforms performance against real-world examples with vulnerabilities in this case being underreported. This indicates that the focus of researchers should be improving detection performance through combining results from tools to enhance detection. 

Finally, recent research focuses on vulnerability detection using machine learning/artificial intelligence approaches. Specifically, the work in [51] discusses vulnerability detection using deep neural networks. The authors highlight the challenges of code representation learning, given the multiple ways in which the same functional statement can be represented by different development styles, and across different languages. Many tools struggle when presented with such non-standard code, and developing methods for analysing it is a key area of research, especially for languages without recognised standard frameworks, such as C and C++. Similar work appears in [52] where the authors attempt to create a taxonomy of vulnerability types, as well as machine learning approaches. Graph-based network learning is also a popular topic in machine learning vulnerability analysis, with [53,54,55,56] discussing work on this topic.

## 3. Methodology

This section provides the proposed threat model, experimental methodology, and metrics used in the analysis.

### 3.1. Threat Model

Our threat model includes an attacker with limited resources. This means that the attacks they mount should not require significant amounts of time and/or computing resources. The attacker is presumed to be technically proficient, but not necessarily an expert. In the following subsections we discuss further assumptions.

#### Access Vectors and Techniques and Tactics

We assume that the attacker is only aware of the specific smart security applications that are installed on the target’s phone. For ease of comparison and analysis, we have assumed that the target is using an Android phone. Many tools exist that allow the export of Android application install files (APKs) from a standard, non-rooted phone. In comparison, exporting installation files from an iPhone can take considerable amounts of time and skill. Finally, we also assume that the attacker and the target are using the same make and model of mobile phone and that the device has not been “rooted.” These assumptions ensure that any vulnerabilities our attacker finds will also exist on the target’s phone. 

The attacks covered in this work are based on the following activities: (a) static analysis of the APK by cloud service Ostorlab; (b) static analysis of the APK by locally hosted service MobSF; and (c) code quality analysis of the decompiled APK using cloud service Snyk. Each of these services was chosen as they offer a completely free tier of service, as well as free access to their JSON API endpoint. This allows for the automation of results collection and analysis. Static analysis is a well-established technique, used by both developers and attackers, to determine potential vulnerabilities. Using a cloud-based tool and a self-hosted one gives the opportunity for comparison between the results obtained. Code quality analysis is used during application implementation by development teams, to ensure that common code vulnerabilities are avoided. Source-code files are analysed for patterns pertaining to specific vulnerabilities, such as path traversals, cross-site request forgeries, hardcoded secrets, and inadequately secure cryptographic algorithms. An attacker can use code quality analysis as a tool to their advantage by decompiling the APK file and discovering even more vulnerabilities. The list of vulnerabilities discovered can then be used by the attacker to target that application on the user’s smartphone.

### 3.2. Experimental Setup

To select a representative set of Android applications for smart locks, a search was made in the Play Store for “smart lock”, “smart security”, “smart camera”, and “smart padlock” applications. We decided to focus on the official application repository (i.e., Play Store) instead of other unofficial ones where users could sideload Android apps, because, as follows: (*i*) we considered that non-security and non-technical savvy users are more likely to use the official application repository and (*ii*) smart device manufacturer often post their companion applications in the official application repository. Fifty-four Android applications, which are listed in Table A1, were downloaded and installed to an Android smartphone running Android 11. The applications selected are companion applications of branded products that fall in the four device categories. These are offered by known manufacturers, such as Master Lock and Schlage, as well as generic products from lesser-known manufacturers. Several of the applications selected are “suite” apps, meaning that they contain the code to control two or more of the devices in the four categories. In addition, applications such as Xiaomi Home and Tuya Smart, and Kasa are designed to control a wide variety of other devices, such as smart lightbulbs and kitchen appliances. These applications have been included in the study as they have an extensive user base [57] and whilst not solely managing smart security products, a significant proportion of the application code is dedicated to supporting these products.

This work used a combination of state-of-art software, both open source and commercially used in SecDevOps, to automatically identify vulnerabilities in Android apps. The identified vulnerabilities were confirmed with manual code inspection by a SecDevOps developer with 10 years of experience. For each application we selected, an APK file was then exported using *AirDroid*. Then, the APK file was automatically scanned against a list of common vulnerabilities for Android apps, which is summarised in Table 1. During the vulnerability scanning, the APKs were uploaded and scanned by *Ostorlab* and *MobSF*. In addition, each APK was decompiled using *jadx* and uploaded to GitHub and analysed by Snyk Code. To avoid including false positives in our results, the results from the different tools were confirmed manually before populating the results summarised in Tables 2–7. The tools used together with their associated URLs can be found in Table A2.

## 4. Experimental Results

This section discusses the results from the analysis of the companion applications of smart locks, as described in Section 3. Firstly, we calculated the mean value and standard deviation for the number of dangerous Android permissions, the number of URLs related to Office of Foreign Assets Control (OFAC) sanctioned nations, and the number of trackers (Table 2). Our analysis suggests that out of the 54 tested apps, the number of dangerous permissions for nine applications is considerably higher than the rest of the group (i.e., one standard deviation higher than the mean, as described in Table 3). As these applications perform essentially the same function with respect to the rest of the tested apps, deviation above the mean suggests unnecessary use of these permissions. This unnecessary use of dangerous permission will increase privacy risk [58], as well as decrease the effort of malicious authors [59]. 

Similar results were found when analysing the number of OFAC Sanction List URLs present inside the 54 tested applications (as shown in Table 3, e.g., in the case of the *Mi Home* application more than 5 standard deviations above the mean). This would indicate that those applications are more likely to share data with servers in countries with poor data privacy laws. Finally, as summarised in Table 2, the number of unique trackers in 11 of the 54 applications exceed the mean by one standard deviation, which suggests that these applications are more likely to be involved in user profiling.

One should note that there is an overlap relating to membership of applications to three sets of results in Table 2. Specifically, the *Mi Home* application is a member of all three groups, whilst the *Nextlock*, *Oaks*, *Sciener*, *Sifely*, and *TTLock* applications are found in two of the groups. As these are all “lesser known” or “generic” apps, our results suggest that for at least these three metrics, branded devices perform better. It also shows that applications that demonstrate significant deviation from average in one metric will do so in others. Nonetheless, the analysis showed that companion applications for branded products are also present in the results, with seven appearing in one of the columns in Table 2. However, none of the applications appear in more than one column, and none appear in the OFAC URL list.

**Table 2:** Dangerous permissions, OFAC URLs, and trackers in companion applications.

Application	No. of Dangerous Permission	No. of OFAC URLs	No. of Trackers
Alarm.com	0	0	4
Arlo	18	0	0
Conexis L1	0	0	5
eGeeTouch	0	0	6
Eufy	21	0	0
Kangaroo	0	0	4
Latch	0	0	4
Mi Home	18	78	7
NextLock	18	29	0
Oaks	20	29	0
Philips EasyKey	18	0	0
Raixer	0	0	4
Schlage Home	0	0	4
Sciener	20	30	0
Sifely	20	29	0
SimpliSafe	0	0	6
SmartSolity	0	0	4
Tedee	0	0	5
TTLock	20	30	0
			
**Mean**	11.81	6.26	2.26
**Standard deviation**	5.26	13.56	1.49

With regards to vulnerabilities from third-party components, we found that 14 out of 54 applications had at least one outdated component included as part of their code. It is worth noting that some of the Common Vulnerabilities and Exposures (CVEs) related to these outdated components are more than 13 years old, e.g., CVE-2010-3492 that is present in six different applications (i.e., *NextLock*, *TTLock*, *Sorex SmartLock*, *Sciener*, *Oaks*, and *Sifely*). Again, these were found in companion applications of “lesser known” devices, suggesting they are more likely to experience this type of vulnerability. Furthermore, our analysis uncovered that each outdated component includes at least one CVE record that is at least a year old, providing attackers with a significant period during which exploits can be developed. We also found that some components relied on deprecated code, such as libraries written in Python version 2.7, which is now out of support and, thus, is not receiving any security updates.

Table 3 contains a list of the vulnerabilities discovered and the application with the largest frequency of that specific vulnerability, as well as the frequency itself. Table 3 also contains the mean number of instances of each vulnerability, as well as the standard deviation. Moreover, Table 4 highlights the companion applications where vulnerabilities are 1, 2, and 3 standard deviations (*σ*) or more above the average for the 54 companion apps, as well as their absolute frequency.

Finally, this work uses two more metrics to assess the security of the companion apps, namely vulnerabilities. The first one is the sum of standard deviations of all vulnerabilities as described in Table 4 plus the standard deviation of the total vulnerabilities. We consider that this sum indicates how poorly, with regards to SecDevOps, a companion application is written, which is reflected by the number of vulnerabilities found compared to the rest of the applications in scope. The second is the vulnerability density, i.e., calculated by the average number of vulnerabilities per MB of decompiled code. As the 54 tested applications vary significantly in size, this metric allows a comparison independent of the size of the app. This metric was preferred instead of a more “traditional metric”, which is the number of lines of source code per vulnerability. This holds as we do not have access to the initial source code, as it was written by the application developer, but rather decompiled code whose reconstruction depends on the decompiler used. Table 5 and Table 6 list the top 10 worst companion applications based on the two aforementioned metrics.

**Table 3:** Vulnerabilities with average frequency and standard deviation.

Vulnerability	Mean	Standard Deviation	Maximum Number of Instances	Application with Most Instances
Path Traversal	11.94	14.91	66	Mi Home
SSRF	15.54	68.28	454	Tapo
Cleartext Transmission	1.48	2.03	9	Tapo
Hardcoded Secrets	23.89	44.04	230	Arlo
SQL Injection	5.30	6.44	34	Mi Home
Inadequate Encryption Strength (TLS v1.0)	5.59	6.69	37	Yale Home 2023
Inadequate AES Padding	3.80	4.56	18	TTLock
Improper Certificate Validation	2.54	3.99	22	Banham Smart Alarm
Broken Cryptographic Algorithm	27.35	40.45	155	Ring

**Table 4:** Snyk identified vulnerabilities per application and deviation from mean.

Application	Path Traversal	SSRF	Cleartext Transmission	Hardcoded Secrets	SQL	TLS v1.0	AES Padding	Cert Validation	Broken Crypto	Total
Arlo				230 (3 *σ*)				14 (2 *σ*)		274 (1 *σ*)
Banham Smart Alarm	34 (1 *σ*)					15 (1 *σ*)		22 (3 *σ*)		
Conexis L1				112 (1 *σ*)					144 (2 *σ*)	280 (1 *σ*)
Hafele					20 (2 *σ*)			8 (1 *σ*)		
Kangaroo						33 (3 *σ*)				
Kasa		240 (3 *σ*)	8 (3 *σ*)					9 (1 *σ*)		392 (2 *σ*)
Lockly								7 (1 *σ*)	103 (1 *σ*)	
Mi Home	66 (3 *σ*)				34 (3 *σ*)	18 (1 *σ*)				274 (1 *σ*)
My Home Alarm			6 (2 *σ*)							
NextLock							13 (2 *σ*)			
Oaks							13 (2 *σ*)			
Philips EasyKey	42 (2 *σ*)						10 (1 *σ*)			
Raixer				106 (1 *σ*)					135 (2 *σ*)	268 (1 *σ*)
Reolink				114 (2 *σ*)				7 (1 *σ*)	149 (3 *σ*)	301 (1 *σ*)
Ring				128 (2 *σ*)				7 (1 *σ*)	155 (3 *σ*)	324 (1 *σ*)
Sciener							13 (2 *σ*)			
Sifely							13 (2 *σ*)			
SimpliSafe				117 (2 *σ*)					95 (1 *σ*)	245 (1 *σ*)
Smart Life	49 (2 *σ*)		4 (1 *σ*)				10 (1 *σ*)			
Sorex SmartLock							10 (1 *σ*)			
Tapo		454 (3 *σ*)	9 (3 *σ*)		24 (2 *σ*)			7 (1 *σ*)		602 (3 *σ*)
TTLock							18 (3 *σ*)			
Tuya Smart	59 (3 *σ*)		4 (1 *σ*)				10 (1 *σ*)			
WeLock							10 (1 *σ*)			
Yale Home 2023				1 *σ*		3 *σ*				
Yale Home	33 (1 *σ*)									
Yale View							10 (1 *σ*)	7 (1 *σ*)		

**Table 5:** Summed standard deviation metric—total standard deviations summed across the 10 metrics in Table 4.

Application	Summed Standard Deviation Metric
Tapo	12
Kasa	9
Mi Home	8
Ring	7
Arlo	6
Reolink	7
Tuya Smart	5
Banham Smart Alarm	5
Yale Home 2023	5

**Table 6:** Vulnerabilities per MB.

Application	Vulnerabilities per MB
Conexis L1	1.70
Yale Home 2023	1.52
Tapo	0.89
Reolink	0.86
Kasa	0.68
Raixer	0.66
Philips EasyKey	0.64
Sorex SmartLock	0.57
Arlo	0.55
TTLock	0.52

## 5. Discussion

Users face two different potential concerns when using any Android app. The first is the security of the application against third-party attackers. These attackers aim to exploit vulnerabilities in an application to gain access to the device and perform actions that the user would not want them to. For instance, this could be transferring money from a bank account, or in the case of companion applications for security devices, disabling an alarm or opening a door. As such, users are at the mercy of application developers with regards to the existence of vulnerabilities in application and keeping them to a minimum to avoid this threat. The second concern relates to the intentions of the application developers themselves. Less scrupulous developers may attempt to harvest data, impairing users’ privacy, for their own use or to sell them to third parties. Examples of this include the use of trackers, communication with suspicious domains, and overuse of dangerous permissions, such as requesting access to the user’s address book or camera. Our analysing showed a significant overlap when one considers the top 20% of applications in the dangerous permissions, OFAC sanctioned countries, and tracker categories. Considering the top nine applications in the first two categories and the top nine in the trackers category, we can see that the Mi Home application appears in all three categories. Oaks, Sciener, Sifely, TTLock, NextLock, and Philips EasyKey applications appear in two of the three categories (see Table 7). There appears to be a strong correlation between excessive use of dangerous permissions and accessing servers in OFAC sanctioned countries, but no correlation between membership of the first two categories and the number of trackers. This might suggest a lower perceived impact of including tracking code in these companion apps. 

**Table 7:** Top 9 apps pertaining to design time decisions.

Dangerous Android Permissions	OFAC Sanctioned URLs	Trackers
Eufy	Mi Home	Mi Home
Oaks	Sciener	eGeeTouch
Sciener	TTLock	SimpliSafe
Sifely	Oaks	Tedee
TTLock	Sifely	Conexis L1
Arlo	NextLock	Alarm.com
Mi Home	Banham Smart Alarm	Latch
NextLock	eSmartLock	SmartSolity
Philips EasyKey	Philips EasyKey	Kangaroo

Use of the standard deviation test clearly indicates which applications contain the most security vulnerabilities compared to their peers. Interestingly, the majority (i.e., 6 of 9) of the applications listed in Table 5 are developed by well-known brands, whilst the remaining 3 are more well-known examples of “generic” brands, namely Mi Home, Reolink, and Tuya Smart. The top two entries in the list, i.e., Kasa and Tapo, are both produced by TP-Link. This suggests that the issues highlighted might be a product of company-wide coding standards, rather than those of individual teams within the organisation. As summarised in Table 6, with respect to the number of vulnerabilities per MB, 6 applications represent well-known brands and 4 represent “generic” products. As discussed earlier, the presence of a substantial number of vulnerabilities, especially ones that are that are published for at least a year, can be considered evidence of poor coding practices and adherence to secure coding standards.

Along with evidence of poor coding practice, there are also indicators of more concerning techniques by several application developers. The correlation between the number of dangerous permissions requested and the number of calls to OFAC sanctioned URLs suggests that the developers at the top of this list are less concerned about limiting access to user data. In general, this would be a concerning result in a review of any application, especially when applications for security devices are concerned as they may contain sensitive data. For example, during our experiments, an unauthenticated Firebase database referenced in the application code was found to contain logs of every device user account configured worldwide. This database included the date, time, and location of every device access. It is worth noting that this data collection is not indicated in the user agreement and as such under data protection laws, such as GDPR, it is unlawful. It is not required for everyday use of the device, and the logical inference is that it has been collected to allow greater targeting in advertising, or for sale to a third party. It should be noted that whilst we only uncovered one instance of this behaviour, we cannot rule out other applications collecting similar intrusive data. It is only because of a coding mistake that we were able to uncover the issue in this instance.

Some of the vulnerabilities found present immediate threats to the user, whilst others would require more subtle exploitation, e.g., via specially crafted phishing messages or drive-by downloads. The first category includes applications with cleartext transmission and hardcoded secrets, which increase the security risk for the end user. Data transmission in plain text is vulnerable to both interception and tampering whilst in transit, whether it is being sent from application to device directly, or via a central management server. Protocol sniffing tools are inexpensive and readily available and can capture and replay traffic from multiple hundreds of meters away, as demonstrated in [1]. In this paper, an unlocking password sent in plain text via Bluetooth was identified for the eGeeTouch smart lock. Once captured, it is then trivial to replay and unlock the device. Similarly, data sent to a central management server must always be encrypted as the intermediary hops are untrusted. 

Hardcoded secrets present significant security concerns when they are used as encryption keys or initialisation strings for cryptographic functions. Another potential concern is user data security and privacy. If the strings found correspond to API keys for online services, then an attacker may be able to gain access to privileged data held remotely and thus breach the user’s privacy. For instance, according to [60], 0.5% of all mobile applications contain Amazon Web Services API keys. In the past, these API keys have been used to access sensitive data and lead to significant breaches, e.g., [61,62]. 

As discussed earlier, another common threat to user privacy that was present in the companion apps of security devices is the use of trackers accompanied by communications sent to OFAC sanctioned countries. Prior work discusses the potential misuse of tracking data by threat actors, for example [63,64]. The Electronic Frontier Foundation has a useful tool that indicates just how much data is collected by web trackers [65]. The more trackers an application contains, the more likely is that the user’s privacy is impaired. When coupled with excessive use of dangerous permissions, an application could exfiltrate personally identifiable information from the user’s device [32,58]. Additionally, received updates may trigger functions based on a user’s location [59]. Whilst this is also possible when data are sent and received from a non-sanctioned source, the OFAC sanctions list indicates locations where such behaviour may be more likely.

Users can take several steps to protect themselves from the vulnerabilities found in this study. Most importantly, they must upgrade their applications whenever an update becomes available. As well as introducing new features, application updates often address vulnerabilities, similar to the ones that were identified in this work. By updating their apps, users assume that they use the “most secure” application version. Nonetheless, as this work suggests, end users are at the mercy of developers with regards to the elimination of vulnerabilities. This holds true as we have identified cases where vulnerabilities that were known for a long time remained in the most recent version (i.e., at the time of our analysis) of some of the companion apps that we have analysed. 

Secondly, Android users are not restricted by Android’s “all or nothing” security model [31], and nowadays can limit the permissions they approve for apps, or revoke permissions of installed apps. Nonetheless, revoking permissions may stop the application from working as expected, or crash it. As a result, users, often non-security and non-technically savvy, are asked to balance the permission requests of a given application against the potential security risk of granting these permissions due to its access to sensitive data and functionality [32,58]. When installing a new app, users are expected to scrutinise the permissions requested, which is not often the case, e.g., as demonstrated in [31,66]. They should also make sure to install the application from a trusted source, such as the Google Play store. Users can also use workarounds in order to protect their security, until an official patch is made available from the application developers. For example, by disabling access to resources, such as Bluetooth and GPS, they can minimise the risk of clear text data transmission between the device and the companion app, and avoid location tracking, respectively. Similarly, as discussed in our previous work [1], end users could disable or avoid the use of functionality offered by the smart security device (e.g., use of tags, use of Bluetooth) to protect themselves from cyber-physical attacks, such as theft, impersonation, and physical injury.

It is worth noting that addressing vulnerabilities of smart security devices relies on three different release cycles, namely for the (*i*) companion application, (*ii*) operating system it runs on, and (*iii*) the firmware of the smart security device hardware. The easiest of all three to update is the application itself, followed by the host operating system, then the physical hardware. The user is therefore at the mercy of three distinct development teams whose update schedules may not align, thus exposing users to a security risk. For instance, it is not uncommon for a device vendor to stop supporting a particular device after a few years, thus depriving users of security updates, which might be available from the OS vendor, as in the case of Android and Samsung devices [67,68]. Furthermore, in comparison with operating system and hardware updates, which usually must be triggered manually, application updates can be configured to auto-install. This removes a burden from the user and ensures that the application itself should be the most secure of all three of the components, as it is the one that can update more frequently. Research does indicate that in some cases users may be reluctant to enable automatic updating, e.g., [69,70]; thus, it is important for smart security device manufacturers to stress the importance of automatic updates.

Static analysis is a challenging task to perform due to the complexity of applications’ source code. The authors have significant experience with state-of-the-art static analysis tools, such as Snyk, Veracode, and SonarQube, which are used in SecDevOps to assess the quality of applications. Such tools look for patterns of behaviour to identify coding issues and potential vulnerabilities that can be exploited by attackers. With modern development frameworks in use across multiple languages, the task of code quality analysis becomes easier, as code is written in the same way, even if the code itself is performing a different functionality. However, when code is written in ways that do not conform to known patterns, code vulnerabilities can become harder to identify by a static analysis tool. The usefulness of results produced by these tools relies on the language used. Analysing newer languages, with more predictable language constructs and well-defined structure, such as Go and Java, produces fewer false positives, whilst “older” languages, such as C and C++, require significant amount of manual investigation to confirm or reject their findings. As such, while the results from static analysis are generally useful, one should note that these tools are prone to false positives and false negatives. Furthermore, in scenarios similar to our work, static analysis becomes more challenging when the Java/Kotlin source code is unavailable. In this case, the Android application must be decompiled prior to static analysis. However, as shown by [71,72], decompilation is not entirely accurate. 

Assessing vulnerabilities using only one tool is not considered good practice. While one tool could give a good indication of the code quality of a given application, it is considered good practice to corroborate their results with at least one more tool, as in this work. As such, as described in Section 3, in this work we have (*i*) used a combination of tools (both open source and commercial) that are often used in SecDevOps and (*ii*) eliminated false positives in the tools’ findings by manually confirming them before recording them in Table 2, Table 3, Table 4, Table 5, Table 6 and Table 7. SAST tools are good at matching known vulnerable coding patterns, but often identify weaknesses that may not be reachable, due to decisions made elsewhere in the code. However, we would argue that the cost of fixing such issues remains low, especially when using the enterprise features of such products, which include the ability to automate pull requests (code updates) for many discovered issues as part of a DevSecOps pipeline. The identified issues *may* not be exploitable at present, but they do indicate poor code quality. A change to other areas of the code may thus render this an exploitable vulnerability in a subsequent version of the application. 

Whilst outside the scope of this work, the usage of dynamic application security testing (DAST) tools, such as [73,74], can validate results found in SAST scans from providers such as Snyk and Ostorlab. They do this through the simulation of both regular user behaviour and that of an attacker to determine the exploitability level of a vulnerability. Other tools such as [75] and the built-in service offered by the Google Play store [76] can provide similar results. A DAST scan can be considered an automated penetration test. When performing a DAST scan, or a traditional manual penetration test, the potential for missing vulnerabilities is considerable. Whilst modern DAST scanners have some default routines built in, they rely on the skill of the operator to determine exactly where to attack and what additional tools to use. In a production environment, the time required to conduct a significant manual investigation of vulnerabilities is limited. Additionally, the skills required to perform a manual penetration test or to correctly configure a DAST scanner are also not widely spread. Automatic remediation of code weaknesses, even when not exploitable, is encouraged for this reason, as well as since it is useful training for developers. For an in-depth discussion of DAST tools in relation to Android application analysis, an interested reader may wish to view [77].

Existing code quality standards may need updating, or new standards created, to ensure that end users are as secure as possible when using a smart security device, such as a smart lock. As we discuss in our previous work [1], the gold standard for products sold in the UK market is the British Standards Institute (BSI) Kite Mark. This is given to products that the institute feels are sufficiently safe and secure for everyday use. However, this does not cover the code quality of the companion application. This is demonstrated by the fact that the companion application for the only BSI certified lock in scope of our analysis was also one of the worst performers when assessed for its code quality. Other standards, such as ISO 9001 [78] and ISO 27001 [79], are used in the promotional literature for some of the devices in scope of this work; however, these are not specifically related to code quality. Finally, one should note that while an ISO standard (i.e., ISO 5055) for software quality exists [80], none of the companion applications assessed in this work appear to conform to it. 

## 6. Conclusions

The use of smart security devices provides the end user with many benefits, such as remote locking and key sharing, but it is not clear that they are adequately aware of the potential risks. The work uncovers that every tested companion application exposes the user to some level of security risk. Most end users who use computers in a work or home setting are aware of basic security issues, such as password reuse, malware, and phishing. Smart security devices introduce a new class of computers into the work and home environment, yet there does not appear to be a similar level of knowledge related to the threats they contain. The end-user mindset is still based on the performance of the” non-smart” predecessors of these devices. Whether a simple key lock, or a wired burglar alarm or CCTV camera, users know approximately the potential threats and can mitigate them. It is unrealistic to expect that those same users possess a deep understanding of code vulnerabilities. However, to truly understand the threats that smart security products contain, this level of insight is required. 

Our work shows that the companion applications expose its users to threats originating from cryptographic vulnerabilities, path traversal, cleartext transmission, SQL injection, and more. Each of these could potentially be used to take control of the companion application, and therefore, also the device it is connected to. We found that both lesser-known and well-known devices possessed significant vulnerabilities. Whilst there are a greater number in the generically branded devices, the well-known devices also possess a significant number of vulnerabilities. In some areas, the well-known brands perform worse than their generic counterparts, for example, in issues per MB of code. None of the applications tested were completely free of vulnerabilities. Three applications showed no identified issues when their source code was scanned with Snyk. However, this may indicate either that there are no vulnerabilities, or that the scanner is unable to recognise the coding style used. Ideally greater transparency of code vulnerabilities should encourage safer design and coding practices. Finally, we consider that when choosing a smart security device, consumers should be confident that the companion applications that interface with it are as secure as they can be. This confidence can be promoted via more transparent vulnerability reporting. Improved static analysis and well-configured dynamic analysis during development and quality assurance could also significantly assist. Moreover, future work could increase the sample size with applications from Google Play and third-party sources, and thus, provide further insight. 

## Figures and Tables

**Table 1:** Vulnerabilities used as security assessment metric of companion apps.

Vulnerabilities	Description	Measure	Tool
Dangerous Android Permissions	Dangerous Android permissions give access to sensitive functions. Excessive use can indicate poor coding practices or attempts to breach user’s privacy.	Number	MobSF
Traffic to OFAC Sanctioned Countries	Office of Foreign Assets Control (OFAC) sanctioned entities are individuals, companies, or nations that the United States government consider to be engaged in either military or economic behaviours to the detriment of the USA and other Western nations. Data transferred to these entities will not have the same level of protection that data processed in the USA and the EU has.	Number	MobSF
Trackers	Trackers can be used to profile users and breach their privacy.	Number	MobSF
Use of outdated vulnerable components	Third-party components with at least one CVE.	Yes/No	Ostorlab
Path traversal	An attacker may gain access to sensitive file locations via incorrect directory permissions.	Number	Snyk
Server Side Request Forgery	SSRF attacks leverage vulnerabilities in code to force an application to serve data that it should not. This can lead to sensitive information disclosure.	Number	Snyk
Cleartext Transmission	Use of insecure protocols such as HTTP. This can lead to information disclosure and person-in-the-middle (PitM) attacks.	Number	Snyk
Hardcoded Secrets	These can be items such as usernames and passwords, or 3rd-party secrets such as API keys.	Number	Snyk
SQL Injection	Running unsanitised SQL commands can lead to breaches in the confidentiality, integrity, and availability.	Number	Snyk
Inadequate Encryption Strength (TLS v1.0)	TLS v1.0 has been deprecated for security reasons, so its use in an application may lead to potential information disclosure attacks.	Number	Snyk
Inadequate Padding for AES Encryption	Where used, AES encryption has the option of several data “padding” methods. Where insecure methods are used, attackers may be able to leverage them to gain system access.	Number	Snyk
Improper Certificate Validation	If HTTPS or other certificates are not correctly validated, fake or self-signed certificates may be used to gain access to secured modes of communication and the data they protect.	Number	Snyk
Broken Cryptographic Algorithms	Use of deprecated methods such as the block cipher mode in AES (CBC) may allow an attacker to use cryptographic techniques to gain unauthorised access to the device.	Number	Snyk

## Data Availability

All APKs used in this work can be downloaded from a dedicated GitHub repository here: https://github.com/aja08379/MDPI_Paper_APKs, accessed on 7 July 2024.

## Appendix A

The following table lists the Android companion applications and their version that are in scope of our work. All APKs can be downloaded from a dedicated GitHub repository here: https://github.com/aja08379/MDPI_Paper_APKs, accessed on 7 July 2024.

**Table A1:** Android applications used in the experiments.

App_Name	Version	Manufacturer
Agoda	8.26.1	Agoda
Alarm.com	5.2	Alarm.com
App Lock	1.0.9	akmobile
Arlo Secure	4.9.1	Arlo
August	23.19.0	August
Blink	6.30.0	Blink
Bold	2.6.5	Bold
cloud smart lock	2.0.2	WeHere
Conexis L1	3.4.13	Yale
Danalock	2.01	Danalock
eSmartLock	4.10.0	ELINK SMART
eufy Security	4.7.6	eufy
EZSET BLE Smart Lock	1.9	EZSET
Hafele Smart Lock	1.3.7	Hafele
Home Assistant	10.2	Home Assistant
igloohome	3.0.4	igloo
Kangaroo	10.33.0	Kangaroo
Kasa	3.3.501	TP-Link
Latch	03.31.00.001	Latch, Inc.
LOCKLY	2.7.1	Lockly
Lokies	0.0.28	Starcom
Master Lock	1.10.1.2	Master Lock
My Home Alarm	3.46	CSL DualCom
Next lock	1.8.6	Safesky
Nuki	10.3	Nuki
Oaks	2.6.0	Oaks
Philips EasyKey Plus	3.9.20	Philips
Raixer	4.2.13	Raixer
Reolink	4.41.0.4.1023	Reolink
Ring	3.64.0	Ring
Schlage Home	4.2.1	Schlage
Sciener	8.0.0	Sciener
Sifely	1.9.0	Sifely
SimpliSafe	5.21.0	SimpliSafe
Smart Life	5.6.1	tuya
Smart Lock	1.2.8	Bauer
Smart Lock	8.1	ttlock
SmartAlarm+2	3.1.2.0627	Banham
SmartLock-Gold	2.1.2	Wenzhou Ouhai Jinjian Hardware Co., Ltd.
SmartSolity	2.0.8	Solity Co.
SOREX SmartLock	1.4.0	Sorex
Tapkey	2.38.6	Tapkey
Tapo	3.0.544	TP-Link
Tapplock	3.9.3	Tapplock Corp.
tedee	1.182.0	tedee
TTLock	7.0.0	TTLock
Tuya Smart	5.6.1	tuya
U-tec	2.1.7.7	U-tec
Verisure	10.2309.166	Verisure
WeLock	4.1.1	WeLock
Xiaomi Home	8.9.706	Xiaomi
Yale Home	6	Yale
Yale Home 2023	3.0.0.75	Yale
Yale View	1.7.4	Yale

The tools used in the paper can be found at the following URLs:

**Table A2:** URLs for the tools used in the experiments.

MobSF	https://github.com/MobSF/Mobile-Security-Framework-MobSF, accessed on 7 July 2024
OstorLab	https://www.ostorlab.co/, accessed on 7 July 2024
Snyk	https://snyk.io/, accessed on 7 July 2024
jadx	https://github.com/skylot/jadx, accessed on 7 July 2024
AirDroid	https://play.google.com/store/apps/details?id=com.sand.airdroid&hl=en_GB, accessed on 7 July 2024
